# Predictive value of early magnetic resonance imaging measures is differentially affected by the dose of interferon beta-1a given subcutaneously three times a week: an exploratory analysis of the PRISMS study

**DOI:** 10.1186/s12883-018-1066-8

**Published:** 2018-05-11

**Authors:** Anthony Traboulsee, David K. B. Li, Mark Cascione, Juanzhi Fang, Fernando Dangond, Aaron Miller

**Affiliations:** 1 University of British Columbia, S113-2211 Wesbrook Mall, Vancouver, BC V6T 1Z7 Canada; 2 Tampa Neurology Associates, South Tampa Multiple Sclerosis Center, 2919 W. Swann Avenue, Suite 401, South Tampa, FL 33609 USA; 3 EMD Serono, Inc., One Technology Place, Rockland, MA 02370 USA; 4 EMD Serono, Inc., 45A Middlesex Turnpike, Billerica, MA 01821 USA; 5 Mount Sinai Hospital, 5 East 98th Street, 1st Floor, New York, NY 10029 USA

**Keywords:** MRI, Multiple sclerosis, T2 lesions, Gadolinium-enhancing lesions, Treatment response, Beta-interferon

## Abstract

**Background:**

On-treatment magnetic resonance imaging lesions may predict long-term clinical outcomes in patients receiving interferon β-1a. This study aimed to assess the effect of active T2 and T1 gadolinium-enhancing (Gd+) lesions on relapses and 3-month confirmed Expanded Disability Status Scale (EDSS) progression in the PRISMS clinical trial.

**Methods:**

Exploratory analyses assessed whether active T2 and T1 Gd + lesions at Month 6, or active T2 lesions at Month 12, predicted clinical outcomes over 4 years in PRISMS.

**Results:**

Mean active T2 lesion number at Month 6 was significantly lower with interferon beta-1a given subcutaneously (IFN β-1a SC) 44 μg and 22 μg 3×/week (tiw) than with placebo (*p* < 0.0001). The presence of ≥4 versus 0 active T2 lesions predicted disability progression at Years 3–4 in the IFN β-1a SC 22 μg group only (*p* < 0.05), whereas the presence of ≥2 versus 0–1 active T2 lesions predicted disability progression in the placebo/delayed treatment (DTx) (Years 2–4; *p* < 0.05) and IFN β-1a SC 22 μg groups (Years 3–4; *p* < 0.05). Greater active T2 lesion number at 6 months predicted relapses in the placebo/DTx group only (≥4 vs. 0, Years 1–4; ≥2 vs. 0–1, Years 2–4; *p* < 0.05), and the presence of T1 Gd + lesions at 6 months predicted disability progression in the IFN β-1a SC 44 μg group only (Year 1; *p* < 0.05). The presence of ≥2 versus 0–1 active T2 lesions at 12 months predicted disability progression over 3 and 4 years in the IFN β-1a SC 44 μg group.

**Conclusion:**

Active T2 lesions at 6 months predicted clinical outcomes in patients receiving placebo or IFN β-1a SC 22 μg, but not in those receiving IFN β-1a SC 44 μg. Active T2 lesions at 12 months may predict outcomes in those receiving IFN β-1a SC 44 μg and are possibly more suggestive of poor response to therapy than T2 results at 6 months.

**Electronic supplementary material:**

The online version of this article (10.1186/s12883-018-1066-8) contains supplementary material, which is available to authorized users.

## Background

Considerable evidence indicates that early treatment of relapsing forms of multiple sclerosis (RMS) is critical in order to delay disease progression and accumulation of irreversible disability. Therefore, as the repertoire of disease-modifying drugs (DMDs) for RMS has grown, it is increasingly important for physicians who treat patients with RMS to know when to consider treatment changes in order to derive maximum benefit from the available treatment options within the available window of opportunity. There is currently no single evidence-based algorithm to guide these decisions due to the inherent heterogeneity of RMS. In its absence, clinical and subclinical indicators of continued disease activity are being evaluated for their ability to define breakthrough disease and potential to predict long-term outcomes.

Randomized clinical trials assessing DMDs for RMS rely on clinical disease endpoints: the occurrence of relapses and confirmed disability progression [1–3]. However, modern MS clinical trial populations show lower levels of clinical disease activity compared with previously reported cohorts [3–6]. The reduced level of disease activity has led to a greater focus on alternative outcomes capable of acting as sensitive surrogates for relapses or disability progression [7–10].

MRI lesions, including T2-weighted hyperintense or T1 gadolinium-enhancing (Gd+) lesions, indicate focal inflammatory activity in RMS and may be present in patients who are clinically stable. These lesions have been proposed as a surrogate marker capable of predicting long-term treatment outcomes in patients with RMS. Although the pathophysiological relationship between MRI lesions and disease progression in MS is complex, MRI is an important component of MS diagnosis, and MRI lesion loads may allow treatment success to be predicted at early time points [9, 11]. Multiple studies of patients treated with interferon (IFN) β-1a therapies have suggested an association between the presence of early on-treatment T2 or T1 Gd + MRI lesions and an increased risk of relapse or disease progression at subsequent time points [7, 9, 10, 12, 13]. MRI results at early time points may, therefore, prove to be a useful tool for assessing the overall efficacy of DMDs, identifying breakthrough disease, and predicting long-term responses of individual patients to therapy.

PRISMS-2 was a 2-year randomized clinical trial designed to evaluate the use of IFN β-1a injected subcutaneously (SC) three times weekly (tiw) in patients with relapsing–remitting MS (RRMS) [3]. Both the 44- and 22-μg doses of IFN β-1a SC tiw demonstrated significant improvements on clinical and MRI measures of disease, compared with placebo [3]. A long-term follow-up study (PRISMS-4), in which patients treated with placebo in the original PRISMS study were reassigned to either IFN β-1a 44 or 22 μg SC tiw, assessed the efficacy of IFN β-1a SC tiw over a 4-year period [14]. Therapy with IFN β-1a SC tiw maintained a treatment benefit after 4 years of treatment, and outcomes were superior in patients who were treated with IFN β-1a SC tiw for all 4 years of the study, compared with those who began IFN β-1a SC tiw treatment after 2 years [14].

This study investigated whether early T2 and T1 Gd + MRI lesions predicted subsequent clinical outcomes in patients treated with IFN β-1a 44 μg SC tiw, IFN β-1a 22 μg SC tiw, or placebo/delayed treatment over a 4-year period in the PRISMS clinical trial.

## Methods

### Study design and treatment

The details of the PRISMS-2 study design have been published previously [3]. Briefly, patients with RRMS, an Expanded Disability Status Scale (EDSS) score of ≤5, and no prior treatment with IFN β were randomized 1:1:1 to IFN β-1a 44 μg SC tiw, IFN β-1a 22 μg SC tiw, or placebo. All patients had proton density (PD)/T2-weighted MRI scans twice a year. A subgroup also had monthly T2 and T1 Gd + scans before and during the first 9 months of treatment (frequent-MRI cohort).

### Exploratory analyses

Exploratory analyses assessed the number of T2 lesions per patient at Month 6 and Month 12. The number of active T2 lesions was calculated as the sum of new, newly enlarging, and recurring T2 lesions on the 6-month MRI scan with reference to the baseline MRI scan. Further exploratory analyses investigated the predictive value of active T2 and T1 Gd + lesions at 6 months on EDSS progression (increase of ≥0.5 points if baseline EDSS was ≥6.0 or increase of ≥1 point if baseline EDSS was < 6.0, confirmed 3 months later) and relapses over Years 1 and 2 (PRISMS-2) and Years 3 and 4 (PRISMS-4). The effect of active T2 lesions at 6 months on time to EDSS progression was calculated over a 4-year period. Additional exploratory analyses evaluated the predictive effect of active T2 lesions at 12 months on EDSS progression and relapses over Years 1, 2, 3, and 4, as well as time to EDSS progression over 4 years.

### Statistical analyses

Between-treatment comparisons of mean active T2 lesion number per patient used a negative binomial regression model with baseline burden of disease and treatment as independent factors. The effect of T2 lesions (≥4 vs. 0 and ≥ 2 vs. 0–1) on relapse and EDSS progression was assessed in the entire cohort using a logistic regression model. The logistic regression models examining the predictive value of T1 Gd + lesions analyzed data from the frequent-MRI cohort. Hazard ratios (HRs) and *p*-values for between-group differences in time to first EDSS progression were calculated using a Cox-proportional hazards model.

## Results

In total, 560 patients were randomized: 187 to the placebo group, 184 to the IFN β-1a 44 μg SC tiw group, and 189 to the IFN β-1a 22 μg SC tiw group. Baseline characteristics were similar among all three treatment groups, as has been previously reported (Table 1) [3]. The proportions of patients who experienced relapses and sustained disability progression by Years 2 and 4 are shown in Table 2**.**

**Table 1:** Demographic characteristics at baseline

	Placebo			IFN β-1a 22 μg SC tiw			IFN β-1a 44 μg SC tiw		
	Number of active T2 lesions at 6 months		All (*n* = 187)	Number of active T2 lesions at 6 months		All (*n* = 189)	Number of active T2 lesions at 6 months		All (*n* = 184)
	0 (*n* = 39)	≥4 (*n* = 64)		0 (*n* = 85)	≥4 (*n* = 28)		0 (*n* = 95)	≥4 (*n* = 24)	
Age, years, mean (SD)	36.4 (7.3)	33.4 (7.8)	34.7 (7.5)	35.6 (6.5)	30.2 (7.3)	34.8 (7.0)	35.5 (8.0)	33.0 (6.7)	35.2 (7.9)
Sex, female, *n* (%)	31 (79)	47 (73)	141 (75)	53 (62)	21 (75)	126 (67)	63 (66)	14 (58)	122 (66)
Race, white, *n* (%)	38 (97)	63 (98)	184 (98)	84 (99)	28 (100)	188 (99)	94 (99)	24 (100)	182 (99)
Time since MS onset, years, mean (SD)	6.6 (5.4)	5.3 (4.3)	6.1 (4.8)	7.9 (6.45)	5.4 (4.3)	7.7 (6.1)	7.1 (5.6)	7.4 (6.9)	7.8 (6.3)
Number of relapses in past 2 years, mean (SD)	2.7 (0.8)	3.3 (1.4)	3.0 (1.3)	2.9 (1.0)	3.2 (1.2)	3.0 (1.1)	2.9 (1.0)	3.5 (1.6)	3.0 (1.1)
EDSS score, median	2.0	2.5	2.5	2.5	2.0	2.5	2.5	3.0	2.5

*EDSS* Expanded Disability Status Scale, *IFN β-1a* interferon beta-1a, *SC* subcutaneously, *SD* standard deviation, *tiw* three times weekly

**Table 2:** Proportion of patients with relapses or confirmed disability progression, Year 2 (PRISMS-2) and 4 (PRISMS-4)

	Placebo/delayed treatment (*n* = 187)	IFN β-1a 22 μg SC tiw (*n* = 189)	IFN β-1a 44 μg SC tiw (*n* = 184)
Relapse by Year 2 (%)	84.5	73.0	67.9
Relapse by Year 4 (%)	90.4	82.0	78.8
Confirmed EDSS progression by Year 2 (%)	39.0	31.2	27.7
Confirmed EDSS progression by Year 4 (%)	48.1	45.0	39.7

EDSS progression was defined as an increase of ≥0.5 points if baseline EDSS was ≥6 or increase of ≥1 point if baseline EDSS was < 6, confirmed 3 months later

*EDSS* Expanded Disability Status Scale, *IFN β-1a* interferon beta-1a, *SC* subcutaneously, *tiw* three times weekly

### Early T2 MRI scan results in PRISMS-2

In total, among subjects with data, 147 of 186 patients (79.0%) in the placebo/delayed treatment group had one or more active T2 lesions at 6 months, compared with 100 of 185 (54.1%) and 87 of 182 (47.8%) patients in the IFN β-1a 22 and 44 μg SC tiw groups, respectively. The mean (standard deviation [SD]) number of active T2 lesions per patient at 6 months was 4.1 (5.72) in the placebo group, compared with 2.1 (4.28) in the IFN β-1a 22 μg SC tiw group and 1.3 (2.34) in the IFN β-1a 44 μg SC tiw group (*p* < 0.0001 for each IFN β-1a group vs. placebo).

### Relationship between active T2 lesions at 6 months and EDSS progression

**Fig. 1:**
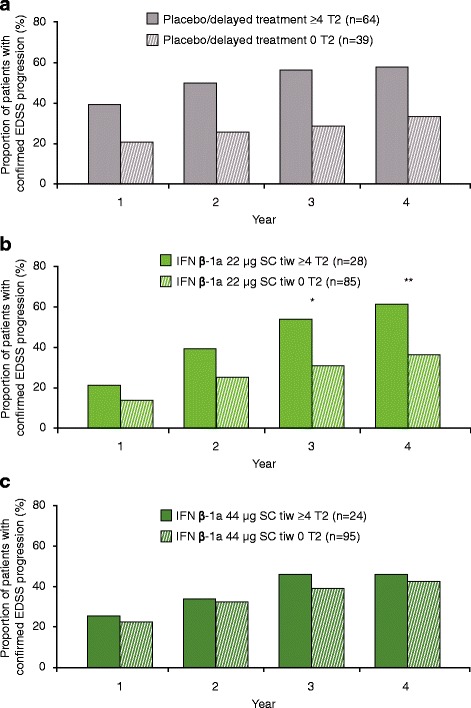
Proportion progressed at each year by ≥4 versus 0 active T2 lesions at 6 months. **a** Placebo/delayed treatment, ≥4 versus 0 T2 lesions at 6 months; **b** IFN β-1a 22 μg SC tiw, ≥4 versus 0 T2 lesions at 6 months; **c** IFN β-1a 44 μg SC tiw, ≥4 versus 0 T2 lesions at 6 months. *p* values indicate differences between patients with differing lesion loads at 6 months within the treatment group. No statistically significant differences were seen in the placebo/delayed treatment or IFN β-1a 44 μg SC tiw group. Values were calculated with a logistic regression model with predictor (≥4 vs. 0 T2 lesions) as a fixed effect; number of relapses within the previous 2 years, age, baseline EDSS score, and baseline burden of disease were independent variables, and *p* values were calculated for the predictive effect of T2 lesion subgroups. *p < 0.05; **p < 0.01. EDSS: Expanded Disability Status Scale; IFN β-1a: interferon beta-1a; SC: subcutaneously; tiw: three times weekly

No statistically significant differences in the proportions of patients with EDSS progression at Years 1, 2, 3, and 4 were seen between patients with ≥4 versus 0 active T2 lesions at Month 6 in the IFN β-1a 44 μg SC tiw or placebo/delayed treatment groups (Fig. 1a and c). However, the presence of ≥4 versus 0 active T2 lesions at Month 6 predicted EDSS progression at Years 3 and 4 in the IFN β-1a 22 μg SC tiw group (Fig. 1b). The presence of ≥2 versus 0–1 active T2 lesions at 6 months predicted EDSS progression in the placebo/delayed treatment (Years 2, 3, and 4) and IFN β-1a 22 μg SC tiw (Years 3 and 4) groups, but not in the IFN β-1a 44 μg SC tiw group (Figure S1a–c shows this in more detail [see Additional file 1]). Notably, EDSS progression in patients treated with IFN β-1a 44 μg SC tiw who had ≥4 or 0 active T2 lesions at Month 6 occurred at a rate similar to that in patients treated with placebo who had no active T2 lesions at Month 6 (Figure S2 shows this in more detail [see Additional file 2]). The predictive performance of active T2 lesions on EDSS progression is shown in Table S1(a) (see Additional file 3). Positive and negative predictive values for EDSS progression according to active T2 lesions at Month 6 (≥4 vs. 0) were consistently higher in the placebo/delayed treatment group than in the IFN β-1a 44 μg SC tiw group.

**Fig. 2:**
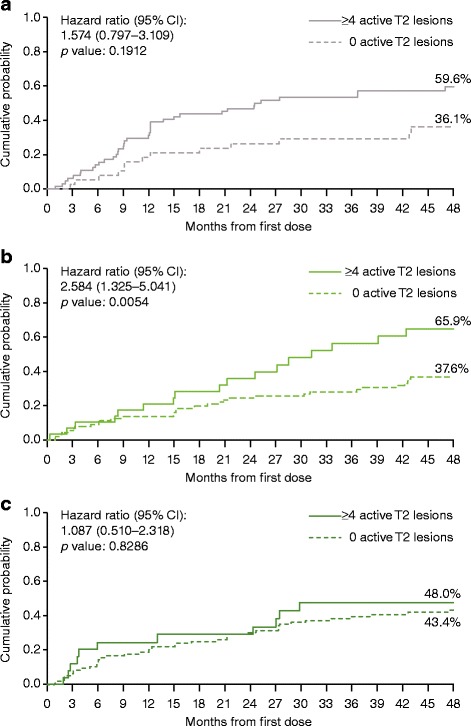
Time to sustained EDSS progression by ≥4 versus 0 active T2 lesions at 6 months. **a** Placebo/delayed treatment group; **b** IFN β-1a 22 μg SC tiw group; **c** IFN β-1a 44 μg SC tiw group. A Cox proportional hazards model, adjusted for number of relapses in the previous 2 years, age, baseline EDSS score, and baseline burden of disease, was used to estimate hazard ratios and *p* values. *p* values are for time to first progression over 4 years. CI: confidence interval; EDSS: Expanded Disability Status Scale; IFN β-1a: interferon beta-1a; SC: subcutaneously; tiw: three times weekly

Time to first EDSS progression over 4 years was significantly reduced for patients with ≥4 versus 0 active T2 lesions at Month 6 in the IFN β-1a 22 μg SC tiw group, but not in the IFN β-1a 44 μg SC tiw or the placebo/delayed treatment group (Fig. 2a–c). For patients with ≥2 versus 0–1 active T2 lesions, significant reductions in time to EDSS progression were seen in the placebo/delayed treatment group (*p* = 0.0132), but not in either IFN β-1a SC tiw group (Figure S3 shows this in more detail [see Additional file 4]).

### Relationship between active T2 lesions at 6 months and relapses

**Fig. 3:**
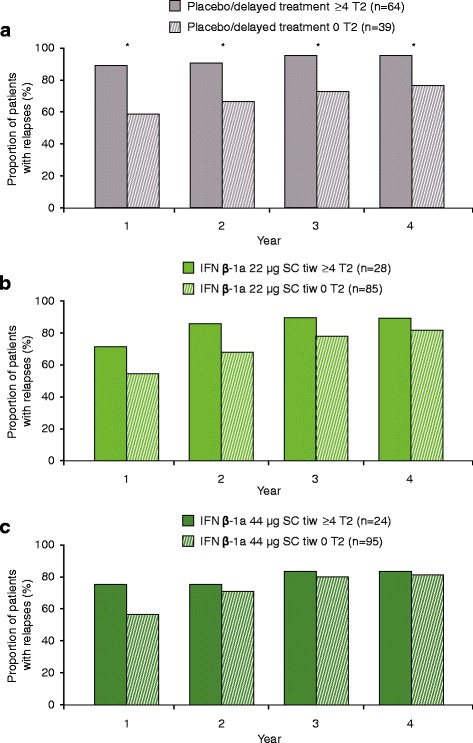
Proportion relapsed at each year: ≥4 versus 0 active T2 lesions at 6 months **a** Placebo/delayed treatment group; **b** IFN β-1a 22 μg SC tiw group; **c** IFN β-1a 44 μg SC tiw group. *p* values indicate differences between patients with differing lesion loads at 6 months within the treatment group. No statistically significant differences were seen in the placebo/delayed treatment or IFN β-1a 44 μg SC tiw group. Values were calculated with a logistic regression model; number of relapses within previous 2 years, age, baseline EDSS score, and baseline burden of disease were independent variables, and *p* values were calculated for the predictive effect of T2 lesion subgroups. *p < 0.05. EDSS: Expanded Disability Status Scale; IFN β-1a: interferon beta-1a; SC: subcutaneously; tiw: three times weekly

The presence of ≥4 versus 0 active T2 lesions at Month 6 predicted relapses in the placebo/delayed treatment group (Years 1–4; Fig. 3a), but not in either IFN β-1a SC tiw group (Fig. 3b and c). Similar results were seen when patients with ≥2 versus 0–1 active T2 lesions at Month 6 were compared (Figure S4 shows this in more detail [see Additional file 5]). The predictive performance of active T2 lesions at 6 months on relapse at Year 2 and Year 4 is shown in Table S1(b) (see Additional file 3).

### Relationship between T1 Gd + lesions at 6 months and clinical outcomes (relapses and EDSS progression)

**Fig. 4:**
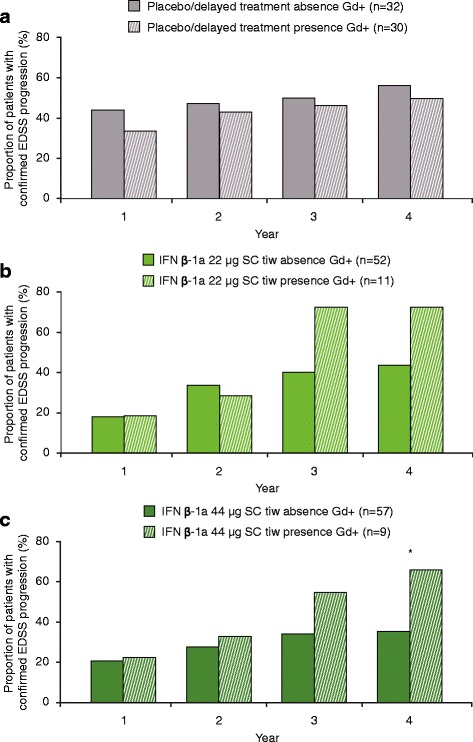
Proportion with EDSS progression at each year by T1 Gd + lesions at 6 months. **a** Placebo/delayed treatment group; **b** IFN β-1a 22 μg SC tiw group; **c** IFN β-1a 44 μg SC tiw group. There was a significant difference in the number of patients who had T1 Gd + lesions at 6 months and who experienced confirmed disability progression at 4 years in the IFN β-1a 44 μg SC tiw group, albeit in a small number of patients (*n* = 9). No statistically significant differences were seen in the placebo/delayed treatment or IFN β-1a 22 μg SC tiw groups. Values were based on logistic regression model adjusting for number of relapses within the previous 2 years, age, baseline EDSS score, and baseline T1 Gd + lesions. *p* values were calculated for the predictive effect of T1 Gd + lesions. *p < 0.05. EDSS: Expanded Disability Status Scale; Gd+: gadolinium-enhancing; IFN β-1a: interferon beta-1a; SC: subcutaneously; tiw: three times weekly

The presence of T1 Gd + lesions at 6 months did not predict EDSS progression in the placebo/delayed treatment group or the IFN β-1a 22 μg SC tiw group in Years 1–4 (Fig. 4a and b). A significantly higher proportion of patients in the IFN β-1a 44 μg SC tiw group who had T1 Gd + lesions at 6 months experienced EDSS progression at Year 4 versus those without T1 Gd + lesions (*p* < 0.05; Fig. 4c). Presumably due to the effectiveness of treatment, the number of patients in the IFN β-1a 44 μg SC tiw group with T1 Gd + lesions at 6 months was small (*n* = 9). However, when comparing patients with ≥2 T1 Gd + lesions versus those with 0–1 T1 Gd + lesions at Month 6, there was a statistically significant difference in the proportion of patients who experienced EDSS progression at Year 2 in the IFN β-1a 44 μg SC tiw group (*p* = 0.0375). Notably, the number of patients in the IFN β-1a 44 μg SC tiw group with ≥2 T1 Gd + lesions at 6 months was very small (*n* = 3).

**Fig. 5:**
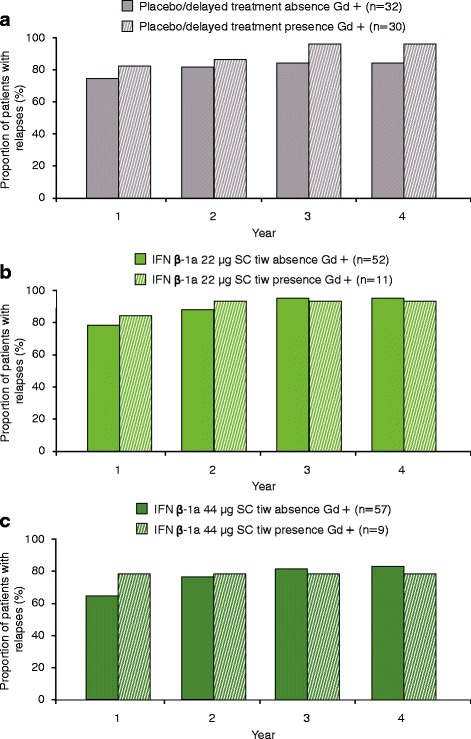
Proportion relapsed at each year by T1 Gd + lesions at 6 months. **a** Placebo/delayed treatment group; **b** IFN β-1a 22 μg SC tiw group; **c** IFN β-1a 44 μg SC tiw group. No statistically significant differences were seen in any of the treatment groups. Values were based on logistic regression model adjusting for number of relapses within the previous 2 years, age, baseline EDSS score, and baseline T1 Gd + lesions. *p* values were calculated for the predictive effect of T1 Gd + lesions. EDSS: Expanded Disability Status Scale; Gd+: gadolinium-enhancing; IFN β-1a: interferon beta-1a; SC: subcutaneously; tiw: three times weekly

The presence of T1 Gd + lesions at 6 months did not predict relapses in any of the treatment groups (Fig. 5a–c). Similar results were seen when comparing patients with ≥2 T1 Gd + lesions versus those with 0–1 T1 Gd + lesions at Month 6 (data not shown).

### Relationship between active T2 lesions at 12 months and EDSS progression

**Fig. 6:**
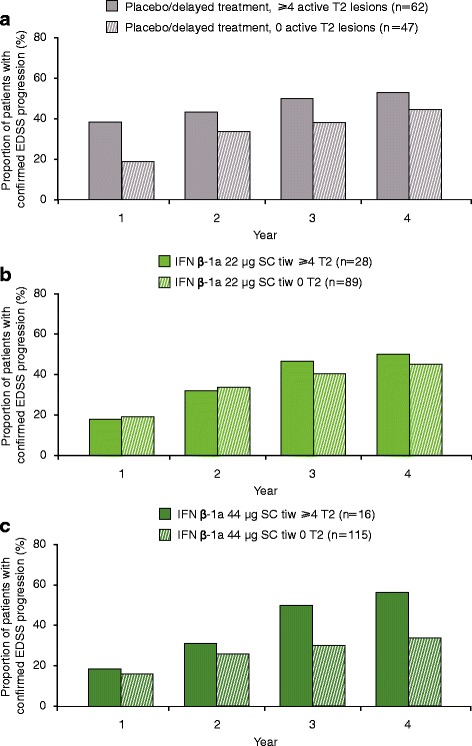
Proportion progressed at each year by ≥4 versus 0 active T2 lesions at 12 months. **a** Placebo/delayed treatment, ≥4 versus 0 T2 lesions at 12 months; **b** IFN β-1a 22 μg SC tiw, ≥4 versus 0 T2 lesions at 12 months; **c** IFN β-1a 44 μg SC tiw, ≥4 versus 0 T2 lesions at 12 months. No statistically significant differences were seen in any treatment group. Values were calculated with a logistic regression model with predictor (≥4 vs 0 T2 lesions) as a fixed effect; number of relapses within the previous 2 years, age, baseline EDSS score, and baseline burden of disease were independent variables, and *p*-values were calculated for the predictive effect of T2 lesion subgroups. EDSS: Expanded Disability Status Scale; IFN β-1a: interferon beta-1a; SC: subcutaneously; tiw: three times weekly

No statistically significant differences in the proportions of patients with EDSS progression at Years 1, 2, 3, and 4 were seen between patients with ≥4 versus 0 active T2 lesions at Month 12 in any of the treatment groups. However, numerically greater proportions of patients with ≥4 versus 0 lesions exhibited EDSS progression over 4 years in each group (Fig. 6). Within the IFN β-1a 44 μg SC tiw group, the numeric difference in proportion with progression between those with ≥4 versus 0 lesions grew more pronounced with each year. The predictive value of active T2 lesions at Month 12 for EDSS progression is shown in Table S1(a) [see Additional file 3]. The presence of ≥2 versus 0–1 active T2 lesions at Month 12 predicted EDSS progression in the IFN β-1a 44 μg SC tiw group over Years 3 and 4 (Figure S5 shows this in more detail [see Additional file 6]).

The presence of ≥4 versus 0 active T2 lesions at 12 months was associated with numeric trends toward reduced time to first EDSS progression in each treatment group (HR: 1.215 [95% confidence interval (CI): 0.634, 2.327], 1.358 [0.707, 2.608], and 1.817 [0.851, 3.879] for placebo/delayed treatment, IFN β-1a 22 μg SC tiw, and IFN β-1a 44 μg SC tiw groups, respectively [*p* > 0.05]). For patients with ≥2 versus 0–1 active T2 lesions, significant reductions in time to EDSS progression were seen in the IFN β-1a 44 μg SC tiw group (HR: 1.970 [95% CI: 1.150, 3.376]; *p* = 0.0136) but not in the placebo/delayed treatment group (*p* = 0.5565) or IFN β-1a 22 μg SC tiw group (*p* = 0.5684).

### Relationship between active T2 lesions at 12 months and relapses

**Fig. 7:**
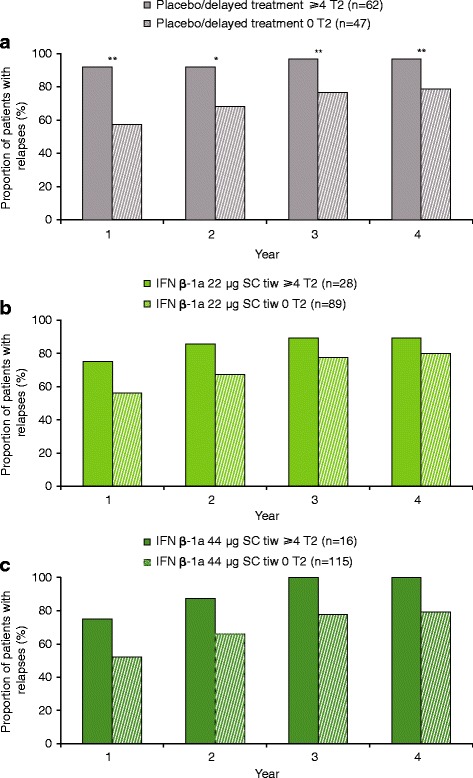
Proportion relapsed at each year: ≥4 versus 0 active T2 lesions at 12 months. **a** Placebo/delayed treatment group; **b** IFN β-1a 22 μg SC tiw group; **c** IFN β-1a 44 μg SC tiw group. *p*-values indicate differences between patients with differing lesion loads at 12 months within the treatment group. No statistically significant differences were seen in the IFN β-1a SC tiw groups. Values were calculated with a logistic regression model; number of relapses within previous 2 years, age, baseline EDSS score, and baseline burden of disease were independent variables, and *p*-values were calculated for the predictive effect of T2-lesion subgroups. *p < 0.05; **p < 0.01. EDSS: Expanded Disability Status Scale; IFN β-1a: interferon beta-1a; SC: subcutaneously; tiw: three times weekly

Similar to the results seen with lesions at Month 6, the presence of ≥4 versus 0 active T2 lesions at Month 12 predicted relapses in the placebo/delayed treatment group (Years 1–4; Fig. 7), but not in either IFN β-1a SC tiw group. Again, results similar to the effect of lesions at 6 months were seen when patients with ≥2 versus 0–1 active T2 lesions at Month 12 were compared (Figure S6 shows this in more detail [see Additional file 7]).

## Discussion

These exploratory analyses demonstrate that active T2 lesions at Month 6 were not predictive of longer term clinical disease activity in the IFN β-1a 44 μg SC tiw group in PRISMS, and were predictive in the placebo/delayed treatment (EDSS progression and relapses) and IFN β-1a 22 μg SC tiw groups (EDSS progression only). Our data both challenge and extend the previous studies that linked the presence of active T2 lesions to suboptimal treatment responses and clinical outcomes [7, 10, 13, 15]. The results of such previous studies led to suggestions that active lesions identified during treatment with IFN β-1a or other DMDs signify treatment failure and should trigger consideration of treatment changes [7, 15]. Our findings instead indicate that early (6 months) active T2 lesions are not predictive of long-term outcomes with high-dose IFN SC tiw, while T1 Gd + lesions at 6 months may be relevant to future clinical outcomes. Additionally, the presence of continued T2 activity after 1 year of treatment suggests that response to treatment is lacking.

Our findings provide new data suggesting that the predictive strength of early on-treatment T2 lesions may be modified by treatment factors. In this historical cohort of patients with RMS, active T2 lesions at 6 months were predictive of clinical outcomes in the placebo/delayed treatment and IFN β-1a 22 μg SC tiw groups, but not in the IFN β-1a 44 μg SC tiw group, suggesting that the predictive strength of early on-treatment MRI lesions may depend on IFN β-1a SC tiw dose. Notably, EDSS progression in the IFN β-1a 44 μg SC tiw group, irrespective of 6-month T2 lesion number, occurred at a rate similar to that in patients in the placebo/delayed treatment group (especially before the treatment switch to IFN β-1a 44 μg SC tiw at the end of Year 2) who had no T2 lesions. Indeed, no trend toward greater likelihood of EDSS progression or relapse was seen in the IFN β-1a SC tiw group, even when patients with ≥4 active T2 lesions at 6 months were compared with patients with no T2 lesions.

Importantly, we also show that on-treatment T1 Gd + lesions at 6 months may be predictive of future disability progression. We propose that the active T2 lesions at 6 months are indicative of accumulated disease activity over the entire period of Months 0–6 when the treatment might not yet be fully effective, while a true lack of treatment response is evident by the T1 Gd + lesion activity at Month 6.

MAGNIMS Consensus Guidelines consider that using a baseline MRI scan as the reference point for new activity after treatment initiation carries the possibility of misinterpretation, as new activity before treatment was initiated or before drug therapy became effective could be mistakenly identified as representing suboptimal response [16]. For instance, in PRISMS-2, the period between baseline and 6 months would include the first 8 weeks of administration, when the dose of IFN β-1a SC tiw was being titrated up to the full assigned dose. Although reduction in MRI activity in the subgroup undergoing monthly scanning was observed as early as 2 months after N β-1a SC tiw treatment began [17], in recognition of the possibility that the full treatment effect may not be evident by 6 months, we also examined as a predictor the new T2 activity at 12 months (with reference to the 6-month scan). The comparison of ≥4 versus 0 T2 lesions at 12 months was not shown to significantly predict future progression. However, numeric trends suggest that lesions at 12 months may be a more useful means of identifying nonresponders to IFN β-1a SC 44 μg than lesions at 6 months; indeed, comparing ≥2 versus 0–1 active T2 lesions at 12 months predicted future progression in this group. Taken together, these observations suggest that T2 activity over the first 6 months of IFN β-1a SC 44 μg treatment may not indicate treatment failure, while activity after a full year is suggestive of nonresponse. Conversely, as the comparison of ≥2 vs 0–1 T2 lesions at 6 months predicted future progression in both the placebo/delayed treatment and IFN β-1a SC 22 μg groups, the IFN β-1a SC 22 μg dose, while beneficial to many patients, shares some similarity with placebo in that results as early as 6 months may be suggestive of future progression.

Previous studies evaluating treatment response according to MRI results in patients receiving IFN β-1a have assessed differing treatment regimens and utilized varying on-treatment MRI time points (6 months to 2 years after study baseline), assessment types (T1 Gd + or T2 lesions), and thresholds for numbers of lesions [8, 10, 12, 18, 19]. As such, the inconsistent results seen between studies suggest that the predictive value of early MRI lesions may depend on IFN β dose, the timing and type of MRI scans obtained, and the overall study protocol. Importantly, a recent study of accrual of long-term disability in a cohort of patients with RMS showed that emergence of new/enlarging T2 lesions in the first 2 years on study did not predict clinical worsening (as measured by EDSS, the Timed-25 Foot Walk, the 9-Hole Peg Test, or the Paced Serial Auditory Addition Test-3) over 10 years, again suggesting that radiological activity alone may not be predictive of long-term outcomes [20].

As the number of effective DMDs available for RMS treatment grows, some experts have advocated a “zero tolerance” approach to subclinical disease activity, suggesting that even a single MRI lesion is evidence of treatment failure and should trigger a change in therapy [21]. However, the results presented in this analysis suggest the need for a more nuanced approach to the prognostic value of on-treatment MRI activity. The predictive nature of T2 lesions is strongly influenced by whether patients are on high-dose, high-frequency treatment at the time when MRI scans are performed, and the presence of a small number of active T2 lesions on early MRI scans should not automatically warrant treatment changes. On the other hand, patients who continue to have T1 Gd + lesions, or active T2 lesions after a year of therapy, may be true non-responders of IFN β-1a SC tiw treatment and might benefit from alternative therapies.

Limitations of this study include the exploratory nature of the analyses and the primary focus on T2 lesions. T1 Gd + lesions were assessed only over 9 months and in just a subset of patients. Data from other studies have suggested that the presence of early T1 Gd + lesions on MRI scans may also be predictive of subsequent clinical outcomes [18].

## Conclusions

The results of this study suggest that although early (6 months) on-treatment active T2 lesions may predict long-term clinical activity in patients receiving placebo or low-dose IFN β-1a SC tiw, this association is not seen in patients treated with high-dose IFN β-1a SC tiw (44 μg). On-treatment T1 Gd + lesions at Month 6, however, may predict subsequent clinical outcomes with IFN β-1a 44 μg SC tiw. Notably, very few patients on treatment have T1 Gd + lesions, indicating that the vast majority of patients respond well to the high-dose IFN β-1a SC tiw treatment. Emergence of active T2 lesions after 6 months of IFN β-1a 44 μg SC tiw treatment in the absence of T1 Gd + lesions should not be cause to consider treatment changes. However, active T2 lesions at 12 months and T1 Gd + lesions at 6 months may be cause to consider nonresponse to treatment. Early MRI results can therefore be successfully integrated into the treatment algorithms that are used to decide whether patients are responding to treatment.

## Additional files

Additional file 1: **Figure S1.** Proportion with EDSS progression at each year by ≥2 versus 0–1 active T2 lesions at 6 months. (a) Placebo/delayed treatment group; (b) IFN β-1a 22 μg SC tiw group; (c) IFN β-1a 44 μg SC tiw group. (PDF 258 kb)

Additional file 2: **Figure S2.** Proportion with EDSS progression at each year in the placebo/delayed treatment and IFN β-1a 44 μg SC tiw groups by ≥4 versus 0 active T2 lesions at 6 months. (PDF 189 kb)

Additional file 3: **Table S1.** (a) Performance of T2 lesions on predicting EDSS progression at Year 2 and Year 4. (b) Performance of T2 lesions on predicting relapse at Year 2 and Year 4. (DOCX 15 kb)

Additional file 4: **Figure S3.** Time to sustained EDSS progression by ≥2 versus 0–1 active T2 lesions at 6 months. (a) Placebo/delayed treatment group; (b) IFN β-1a 22 μg SC tiw group; (c) IFN β-1a 44 μg SC tiw group. (PDF 229 kb)

Additional file 5: **Figure S4.** Proportion relapsed at each year by ≥2 versus 0–1 active T2 lesions at 6 months. (a) Placebo/delayed treatment group; (b) IFN β-1a 22 μg SC tiw group; (c) IFN β-1a 44 μg SC tiw group. (PDF 269 kb)

Additional file 6: **Figure S5.** Proportion progressed at each year by ≥2 versus 0–1 active T2 lesions at 12 months. (a) Placebo/delayed treatment, ≥2 versus 0–1 T2 lesions at 12 months; (b) IFN β-1a 22 μg SC tiw, ≥2 versus 0–1 T2 lesions at 12 months; (c) IFN β-1a 44 μg SC tiw, ≥2 versus 0–1 T2 lesions at 12 months. (PDF 183 kb)

Additional file 7: **Figure S6.** Proportion relapsed at each year by ≥2 versus 0–1 active T2 lesions at 12 months. (a) Placebo/delayed treatment group; (b) IFN β-1a 22 μg SC tiw group; (c) IFN β-1a 44 μg SC tiw group. (PDF 273 kb)

## Abbreviations

CI: Confidence interval
DMD: Disease-modifying drug
EDSS: Expanded Disability Status Scale
Gd +: Gadolinium-enhancing
HR: Hazard ratio
IFN: Interferon
MRI: Magnetic resonance imaging
MS: Multiple sclerosis
PD: Proton density
RMS: Relapsing forms of multiple sclerosis
RRMS: Relapsing–remitting multiple sclerosis
SC: Subcutaneously
SD: Standard deviation
tiw: Three times weekly
Y: Year
