# Supplementary material for: Predictive value of early magnetic resonance imaging measures is differentially affected by the dose of interferon beta-1a given subcutaneously three times a week: an exploratory analysis of the PRISMS study
Source: BMC Neurol. 2018 May 11;18:68. doi: 10.1186/s12883-018-1066-8 (PMC5946401; doi:10.1186/s12883-018-1066-8)
Supplement: Supplementary file 1 — Figure S1. Proportion with EDSS progression at each year by ≥2 versus 0–1 active T2 lesions at 6 months. (a) Placebo/delayed treatment group; (b) IFN β-1a 22 μg SC tiw group; (c) IFN β-1a 44 μg SC tiw group. (PDF 258 kb) [file 12883_2018_1066_MOESM1_ESM.pdf]

Additional file 1

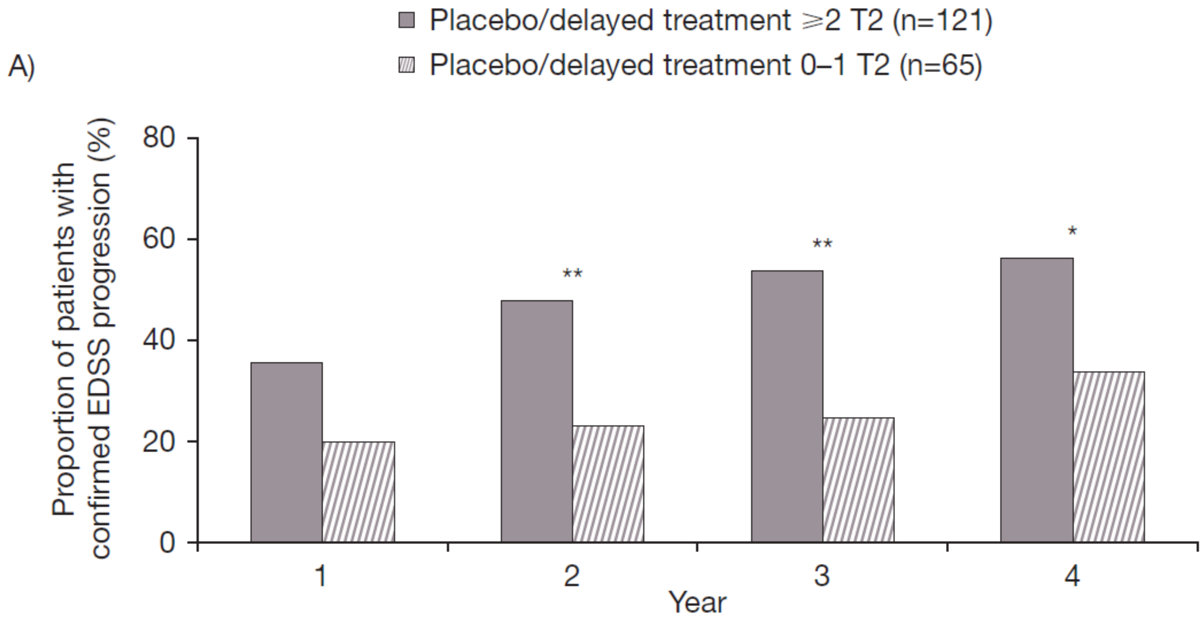

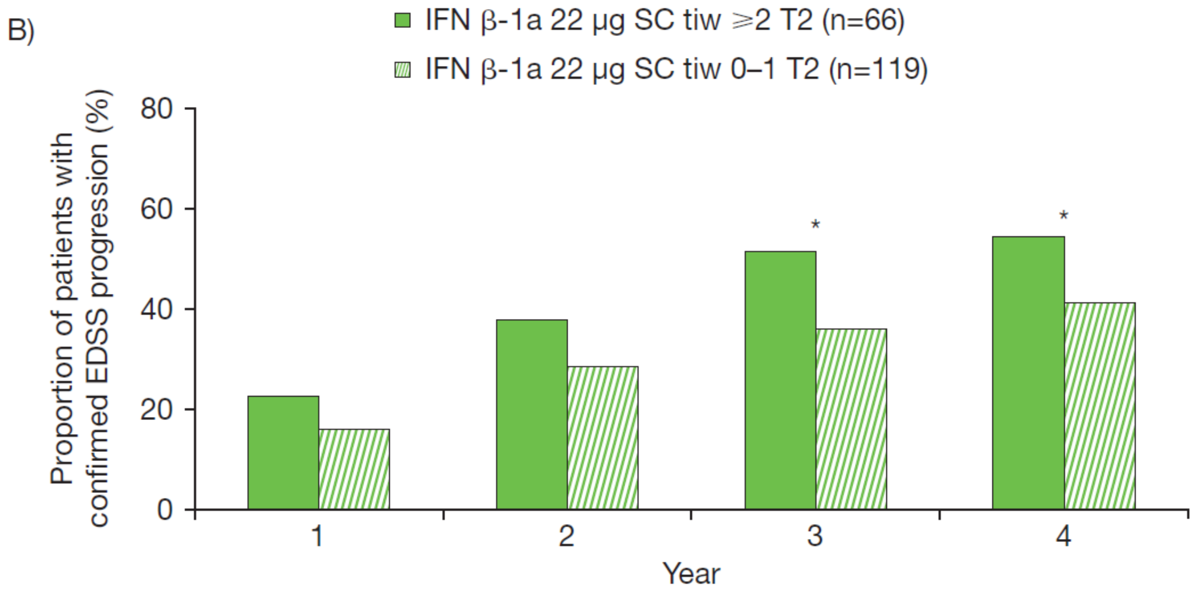

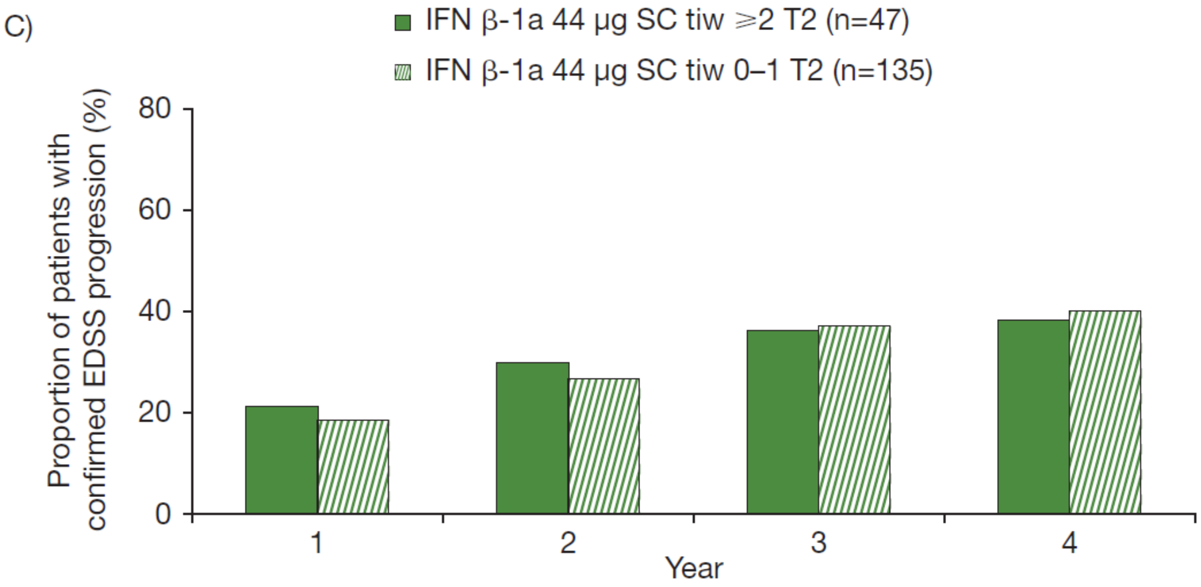

Supplementary Fig. 1 Proportion with EDSS progression at each year by ≥2 versus 0–1 active T2 lesions at 6 months.

(a) Placebo/delayed treatment group; (b) IFN β-1a 22 μg SC tiw group; (c) IFN β-1a 44 μg SC tiw group. *p* values indicate differences between patients with differing lesion loads at 6 months within the treatment group. No statistically significant differences were seen in the IFN β-1a 44 μg SC tiw group. Values were calculated with a logistic regression model with predictor (≥2 vs. 0–1 T2 lesions) as a fixed effect; number of relapses within previous 2 years, age, baseline EDSS score, and baseline burden of disease were independent variables, and *p* values were calculated for the predictive effect of T2 lesion subgroups.

*p<0.05; **p<0.01.

EDSS: Expanded Disability Status Scale; IFN β-1a: interferon beta-1a; SC: subcutaneously; tiw: three times weekly.
