# Supplementary material for: Predictive value of early magnetic resonance imaging measures is differentially affected by the dose of interferon beta-1a given subcutaneously three times a week: an exploratory analysis of the PRISMS study
Source: BMC Neurol. 2018 May 11;18:68. doi: 10.1186/s12883-018-1066-8 (PMC5946401; doi:10.1186/s12883-018-1066-8)
Supplement: Supplementary file 2 — Figure S2. Proportion with EDSS progression at each year in the placebo/delayed treatment and IFN β-1a 44 μg SC tiw groups by ≥4 versus 0 active T2 lesions at 6 months. (PDF 189 kb) [file 12883_2018_1066_MOESM2_ESM.pdf]

Additional file 2

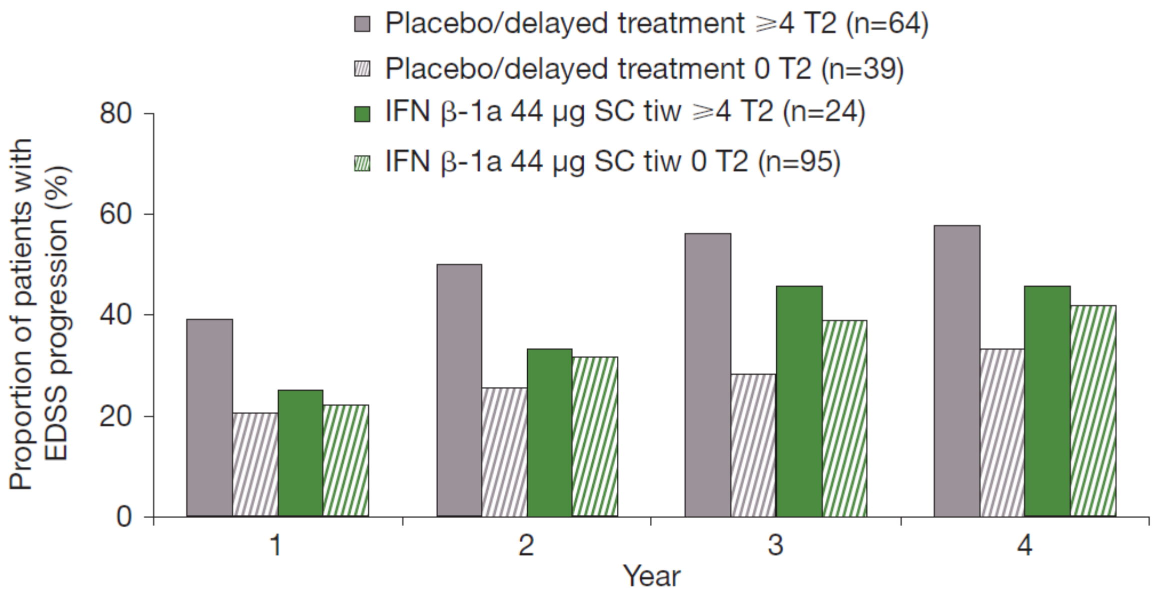

Supplementary Fig. 2 Proportion with EDSS progression at each year in the placebo/delayed treatment and IFN β-1a 44 μg SC tiw groups by ≥4 versus 0 active T2 lesions at 6 months.

EDSS: Expanded Disability Status Scale; IFN β-1a: interferon beta-1a; SC: subcutaneously; tiw: three times weekly.
