# Supplementary material for: Predictive value of early magnetic resonance imaging measures is differentially affected by the dose of interferon beta-1a given subcutaneously three times a week: an exploratory analysis of the PRISMS study
Source: BMC Neurol. 2018 May 11;18:68. doi: 10.1186/s12883-018-1066-8 (PMC5946401; doi:10.1186/s12883-018-1066-8)
Supplement: Supplementary file 3 — Table S1. (a) Performance of T2 lesions on predicting EDSS progression at Year 2 and Year 4. (b) Performance of T2 lesions on predicting relapse at Year 2 and Year 4. (DOCX 15 kb) [file 12883_2018_1066_MOESM3_ESM.docx]

**Additional file 3**

**Supplementary Table 1.** (a) Performance of T2 lesions on predicting EDSS progression at Year 2 and Year 4.

|  | Positive predictive value (%) | Negative predictive value (%) | Sensitivity (%) | Specificity (%) |
| --- | --- | --- | --- | --- |
| EDSS progression at Year 2, IFN β-1a 44 μg SC tiw | | | | |
| 0–1 vs. ≥2 T2 lesions at 6 months | 29.8 | 73.3 | 28.0 | 75.0 |
| 0 vs. ≥4 T2 lesions at 6 months | 33.3 | 68.4 | 21.1 | 80.2 |
| 0–1 vs. ≥2 T2 lesions at 6 months | 35.5 | 73.8 | 22.0 | 84.6 |
| 0 vs. ≥4 T2 lesions at 12 months | 31.3 | 73.9 | 14.3 | 88.5 |
| EDSS progression at Year 2, placebo group | | | | |
| 0–1 vs. ≥2 T2 lesions at 6 months | 47.9 | 76.9 | 79.5 | 44.2 |
| 0 vs. ≥4 T2 lesions at 6 months | 50.0 | 74.4 | 76.2 | 47.5 |
| 0–1 vs. ≥2 T2 lesions at 12 months | 43.8 | 64.9 | 63.9 | 44.9 |
| 0 vs. ≥4 T2 lesions at 12 months | 43.5 | 66.0 | 62.8 | 47.0 |
| EDSS progression at Year 4, IFN β-1a 44 μg SC tiw | | | | |
| 0–1 vs. ≥2 T2 lesions at 6 months | 38.3 | 60.0 | 25.0 | 73.6 |
| 0 vs. ≥4 T2 lesions at 6 months | 45.8 | 57.9 | 21.6 | 80.9 |
| 0–1 vs. ≥2 T2 lesions at 12 months | 61.3 | 64.4 | 26.4 | 88.9 |
| 0 vs. ≥4 T2 lesions at 12 months | 56.3 | 66.1 | 18.8 | 91.6 |
| EDSS progression at Year 4, placebo/delayed treatment | | | | |
| 0–1 vs. ≥2 T2 lesions at 6 months | 56.2 | 66.2 | 75.6 | 44.8 |
| 0 vs. ≥4 T2 lesions at 6 months | 57.8 | 66.7 | 74.0 | 49.1 |
| 0–1 vs. ≥2 T2 lesions at 12 months | 53.3 | 55.4 | 62.9 | 45.6 |
| 0 vs. ≥4 T2 lesions at 12 months | 53.2 | 55.3 | 61.1 | 47.3 |

Positive predictive value is the proportion of patients in the category with the higher number of active T2 lesions (≥2 or ≥4) who have EDSS progression (at Year 2 or Year 4). Negative predictive value is the proportion of patients in the category with the lower number of active T2 lesions (0–1 or 0) who do not have EDSS progression (at Year 2 or Year 4).

EDSS: Expanded Disability Status Scale; IFN β-1a: interferon beta-1a; SC: subcutaneously; tiw: three times weekly.

**Supplementary Table 1.** (b) Performance of T2 lesions on predicting relapse at Year 2 and Year 4.

|  | Positive predictive value (%) | Negative predictive value (%) | Sensitivity (%) | Specificity (%) |
| --- | --- | --- | --- | --- |
| Relapse at Year 2, IFN β-1a 44 μg SC tiw | | | | |
| 0–1 vs. ≥2 T2 lesions at 6 months | 74.5 | 34.1 | 28.2 | 79.3 |
| 0 vs. ≥4 T2 lesions at 6 months | 75.0 | 29.5 | 21.2 | 82.4 |
| 0–1 vs. ≥2 T2 lesions at 12 months | 80.6 | 34.9 | 20.5 | 89.7 |
| 0 vs. ≥4 T2 lesions at 12 months | 87.5 | 33.9 | 15.6 | 95.1 |
| Relapse at Year 2, placebo | | | | |
| 0–1 vs. ≥2 T2 lesions at 6 months | 89.3 | 24.6 | 68.8 | 55.2 |
| 0 vs. ≥4 T2 lesions at 6 months | 90.6 | 33.3 | 69.0 | 68.4 |
| 0–1 vs. ≥2 T2 lesions at 12 months | 90.5 | 23.0 | 62.5 | 63.0 |
| 0 vs. ≥4 T2 lesions at 12 months | 91.9 | 31.9 | 64.0 | 75.0 |
| Relapse at Year 4, IFN β-1a 44 μg SC tiw | | | | |
| 0–1 vs. ≥2 T2 lesions at 6 months | 83.0 | 22.2 | 27.1 | 78.9 |
| 0 vs. ≥4 T2 lesions at 6 months | 83.3 | 18.9 | 20.6 | 81.8 |
| 0–1 vs. ≥2 T2 lesions at 12 months | 90.3 | 23.5 | 19.7 | 92.1 |
| 0 vs. ≥4 T2 lesions at 12 months | 100.0 | 20.9 | 15.0 | Undefined |
| Relapse at Year 4, placebo/delayed treatment | | | | |
| 0–1 vs. ≥2 T2 lesions at 6 months | 83.0 | 22.2 | 27.1 | 78.9 |
| 0 vs. ≥4 T2 lesions at 6 months | 95.3 | 23.1 | 67.0 | 75.0 |
| 0–1 vs. ≥2 T2 lesions at 12 months | 95.2 | 16.2 | 61.7 | 70.6 |
| 0 vs. ≥4 T2 lesions at 12 months | 96.8 | 21.3 | 61.9 | 83.3 |

Positive predictive value is the proportion of patients in the category with the higher number of active T2 lesions (≥2 or ≥4) who have relapses (at Year 2 or Year 4).
Negative predictive value is the proportion of patients in the category with the lower number of active T2 lesions (0–1 or 0) who do not have relapses (at Year 2 or Year 4).

EDSS: Expanded Disability Status Scale; IFN β-1a: interferon beta-1a; SC: subcutaneously; tiw: three times weekly.
