# Supplementary material for: Predictive value of early magnetic resonance imaging measures is differentially affected by the dose of interferon beta-1a given subcutaneously three times a week: an exploratory analysis of the PRISMS study
Source: BMC Neurol. 2018 May 11;18:68. doi: 10.1186/s12883-018-1066-8 (PMC5946401; doi:10.1186/s12883-018-1066-8)
Supplement: Supplementary file 4 — Figure S3. Time to sustained EDSS progression by ≥2 versus 0–1 active T2 lesions at 6 months. (a) Placebo/delayed treatment group; (b) IFN β-1a 22 μg SC tiw group; (c) IFN β-1a 44 μg SC tiw group. (PDF 229 kb) [file 12883_2018_1066_MOESM4_ESM.pdf]

Additional file 4

A)

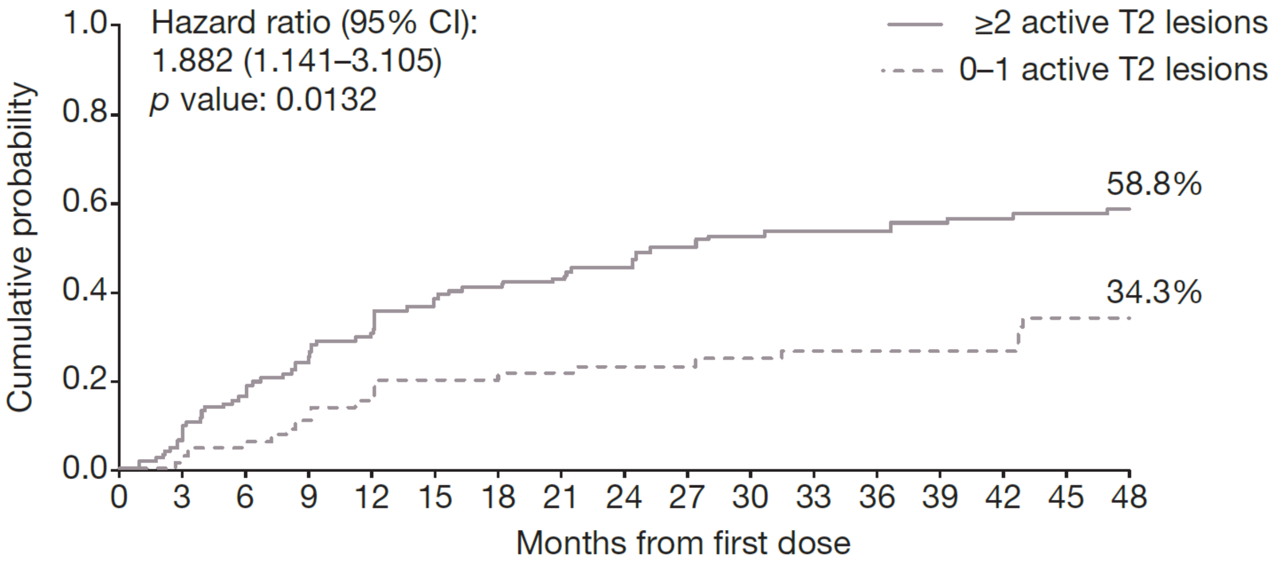

B)

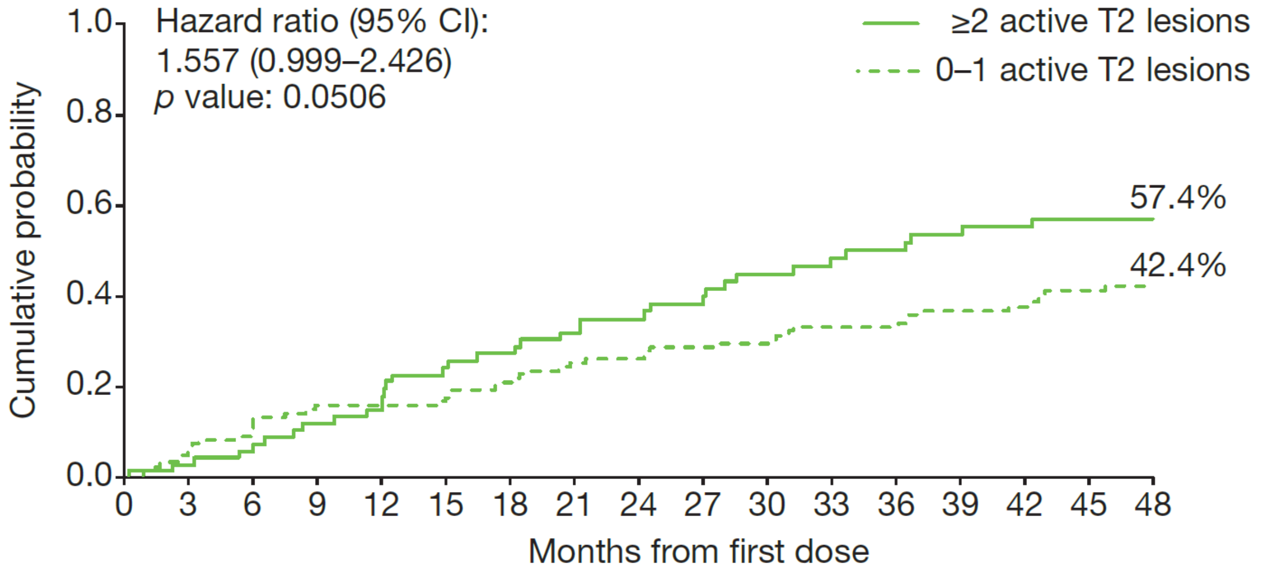

C)

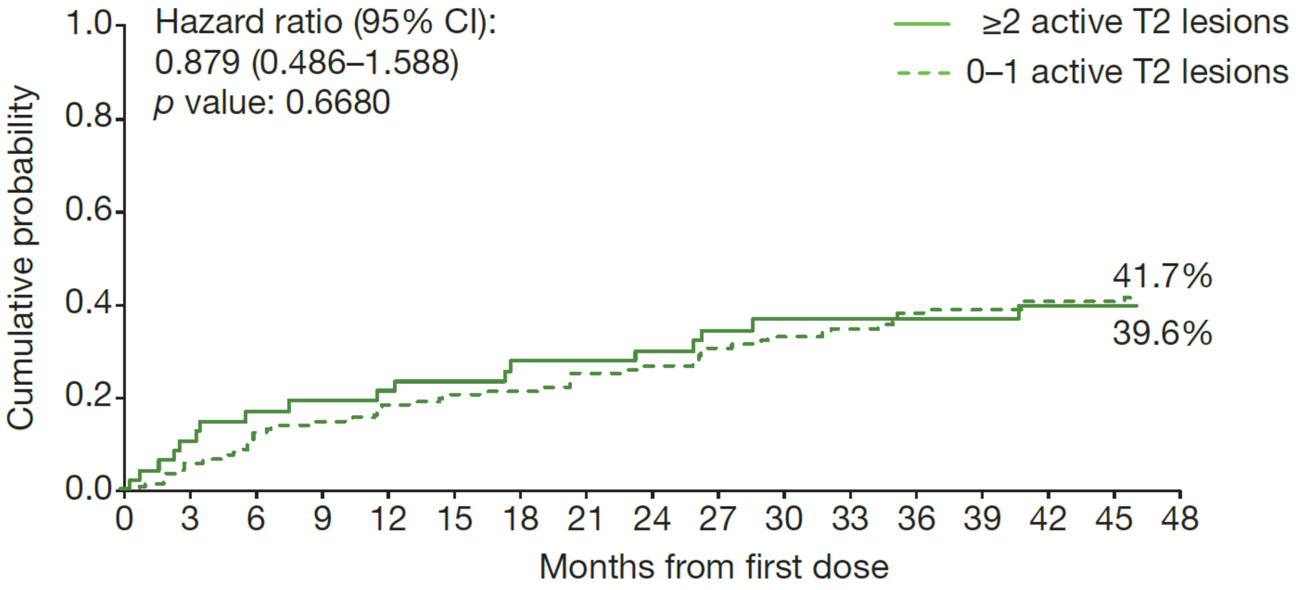

Supplementary Fig. 3 Time to sustained EDSS progression by ≥2 versus 0–1 active T2 lesions at 6 months.

(a) Placebo/delayed treatment group; (b) IFN β-1a 22 µg SC tiw group; (c) IFN β-1a 44 µg SC tiw group.

A Cox proportional hazards model, adjusted for number of relapses in the previous 2 years, age, baseline EDSS score, and baseline burden of disease, was used to estimate hazard ratios and *p* values. *p* values are for time to first progression over 4 years.

CI: confidence interval; EDSS: Expanded Disability Status Scale; IFN β-1a: interferon beta-1a; SC: subcutaneously; tiw: three times weekly.
