# Supplementary material for: Predictive value of early magnetic resonance imaging measures is differentially affected by the dose of interferon beta-1a given subcutaneously three times a week: an exploratory analysis of the PRISMS study
Source: BMC Neurol. 2018 May 11;18:68. doi: 10.1186/s12883-018-1066-8 (PMC5946401; doi:10.1186/s12883-018-1066-8)
Supplement: Supplementary file 6 — Figure S5. Proportion progressed at each year by ≥2 versus 0–1 active T2 lesions at 12 months. (a) Placebo/delayed treatment, ≥2 versus 0–1 T2 lesions at 12 months; (b) IFN β-1a 22 μg SC tiw, ≥2 versus 0–1 T2 lesions at 12 months; (c) IFN β-1a 44 μg SC tiw, ≥2 versus 0–1 T2 lesions at 12 months. (PDF 183 kb) [file 12883_2018_1066_MOESM6_ESM.pdf]

Additional file 6

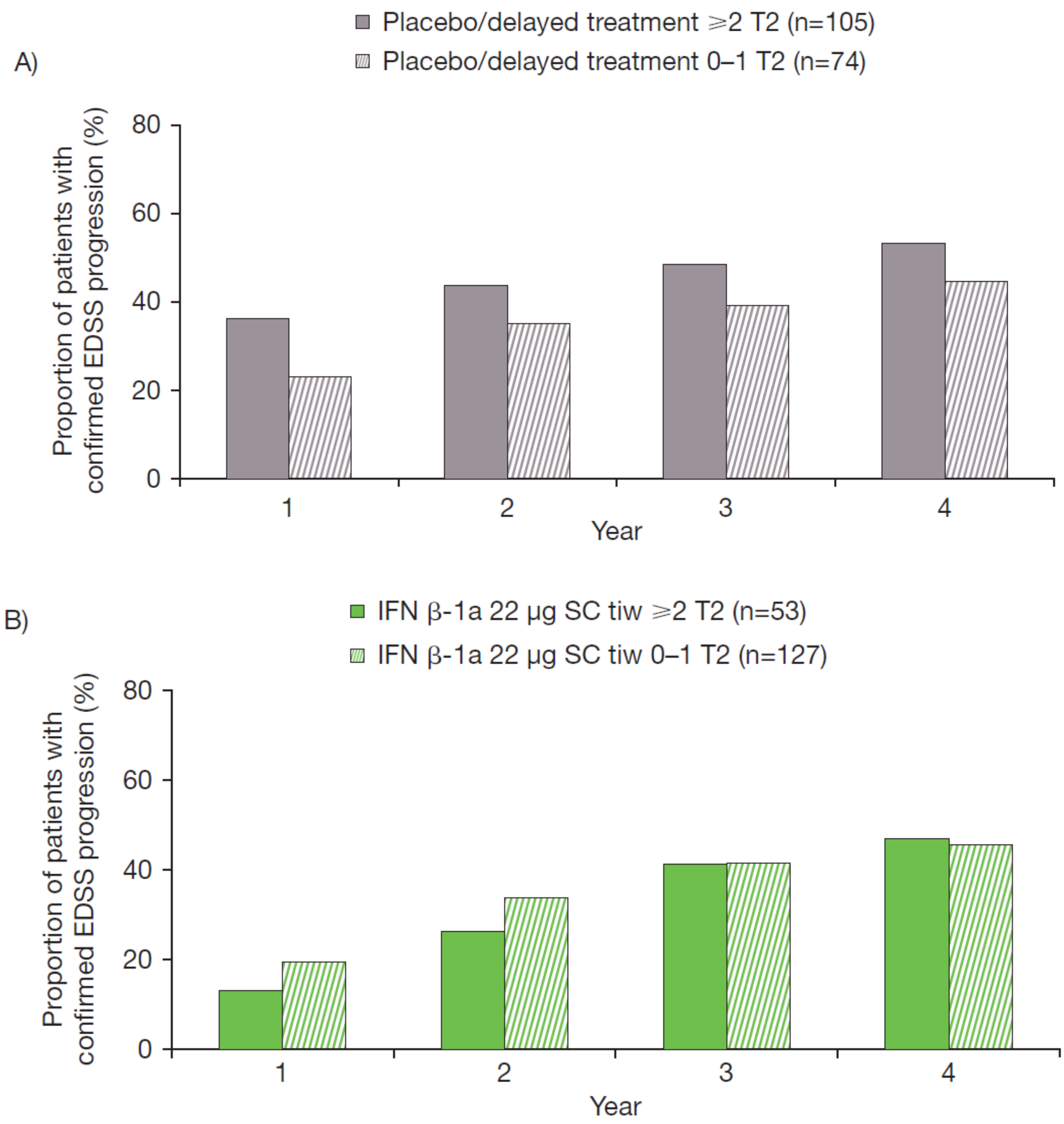

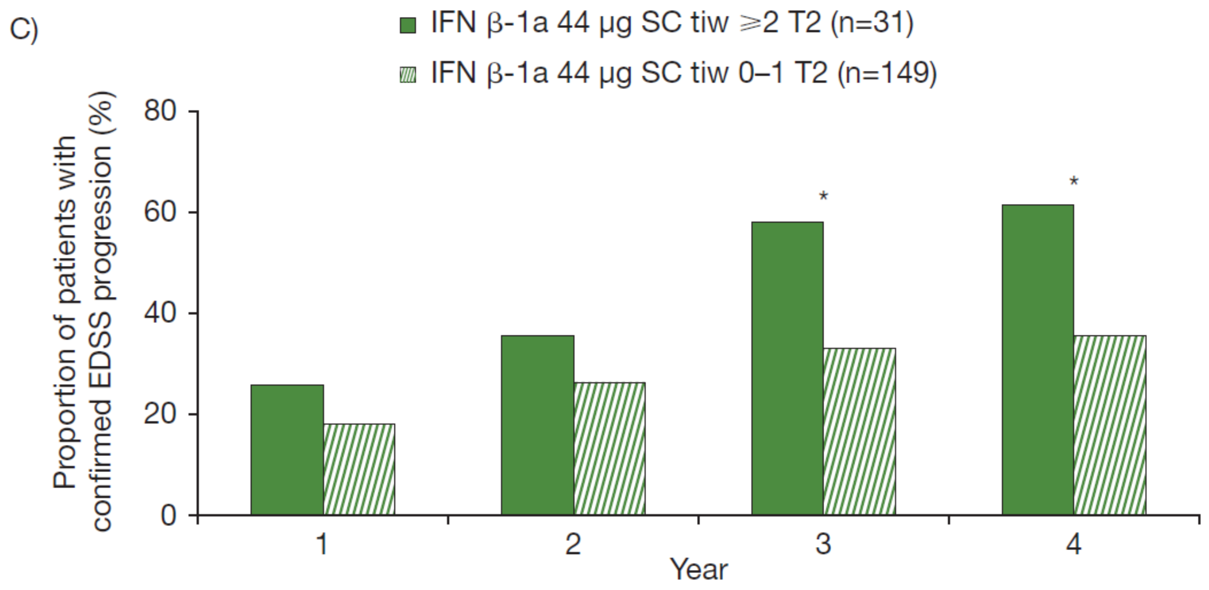

Supplementary Fig. 5 Proportion progressed at each year by ≥2 versus 0–1 active T2 lesions at 12 months.

(a) Placebo/delayed treatment, ≥2 versus 0–1 T2 lesions at 12 months; (b) IFN β-1a 22 µg SC tiw, ≥2 versus 0–1 T2 lesions at 12 months; (c) IFN β-1a 44 µg SC tiw, ≥2 versus 0–1 T2 lesions at 12 months. *p*-values indicate differences between patients with differing lesion loads at 12 months within the treatment group. No statistically significant differences were seen in the placebo/delayed treatment or IFN β-1a 22 µg SC tiw groups. Values were calculated with a logistic-regression model with predictor (≥2 vs. 0–1 T2 lesions) as a fixed effect; number of relapses within the previous 2 years, age, baseline EDSS score, and baseline burden of disease were independent variables, and *p*-values were calculated for the predictive effect of T2 lesion subgroups.

*$p<0.05$.

EDSS: Expanded Disability Status Scale; IFN β-1a: interferon beta-1a; SC: subcutaneously; tiw: three times weekly.
